# Efficacy and limitations of ChatGPT as a biostatistical problem-solving tool in medical education in Serbia: a descriptive study

**DOI:** 10.3352/jeehp.2023.20.28

**Published:** 2023-10-16

**Authors:** Aleksandra Ignjatović, Lazar Stevanović

**Affiliations:** 1 Department of Medical Statistics and Informatics, Faculty of Medicine, University of Niš, Niš, Serbia; 2 Faculty of Medicine, University of Niš, Niš, Serbia; Hallym University, Korea

**Keywords:** Artificial intelligence, Biostatistics, Medical education, Reproducibility of results

## Abstract

**Purpose:**

This study aimed to assess the performance of ChatGPT (GPT-3.5 and GPT-4) as a study tool in solving biostatistical problems and to identify any potential drawbacks that might arise from using ChatGPT in medical education, particularly in solving practical biostatistical problems.

**Methods:**

ChatGPT was tested to evaluate its ability to solve biostatistical problems from the *Handbook of Medical Statistics* by Peacock and Peacock in this descriptive study. Tables from the problems were transformed into textual questions. Ten biostatistical problems were randomly chosen and used as text-based input for conversation with ChatGPT (versions 3.5 and 4).

**Results:**

GPT-3.5 solved 5 practical problems in the first attempt, related to categorical data, cross-sectional study, measuring reliability, probability properties, and the t-test. GPT-3.5 failed to provide correct answers regarding analysis of variance, the chi-square test, and sample size within 3 attempts. GPT-4 also solved a task related to the confidence interval in the first attempt and solved all questions within 3 attempts, with precise guidance and monitoring.

**Conclusion:**

The assessment of both versions of ChatGPT performance in 10 biostatistical problems revealed that GPT-3.5 and 4’s performance was below average, with correct response rates of 5 and 6 out of 10 on the first attempt. GPT-4 succeeded in providing all correct answers within 3 attempts. These findings indicate that students must be aware that this tool, even when providing and calculating different statistical analyses, can be wrong, and they should be aware of ChatGPT’s limitations and be careful when incorporating this model into medical education.

## Graphical abstract

[Fig f1-jeehp-20-28]

## Introduction

### Background/rationale

The Chat Generator Pre-Trained Transformer (ChatGPT), a language model created and released on November 30, 2022, by OpenAI (https://openai.com/), is all-pervasive and has many potential uses in many different fields, including content creation, translation of languages, text summarization, educational assistance, creative writing, programming, learning a new language, data evaluation, and interactions with other people [1]. GPT-4 appeared on March 14, 2023, by OpenAI. After that, the original ChatGPT was renamed as GPT-3.5. Therefore, in this article, ChatGPT comprises GPT-3.5 and GPT-4.

ChatGPT is made to interact with users naturally and logically, with responses that are frequently indistinguishable from a human-produced text. Because ChatGPT can answer in several languages and produce sophisticated, highly developed responses based on advanced modeling, it is superior to its GPT-based forerunners. The large language model, a type of artificial intelligence (AI), has been trained on vast data and can produce remarkably accurate and human-like prose. A model can store more data and execute more complicated tasks if it has more parameters [2]. ChatGPT more accurately simulates human speech than any previous computer program. It can substitute for a real discussion partner and provide unexpectedly good responses to requests and complex data [3]. Concerns have been expressed about potential bias based on the datasets used for ChatGPT training, which can restrict its functionality and may produce factual errors. Despite its exceptional capabilities, ethical issues have dogged generative AI. There have been ongoing discussions about who owns the huge amounts of data that are available online. Additionally, as these tools develop, it becomes more difficult to distinguish between human and algorithmic creations [1].

ChatGPT has also demonstrated promise in many natural language processing activities, including medical education and the production of multiple-choice questions [2]. Early research has suggested that ChatGPT can provide accurate information for medical students in certain domains, but more research is needed to establish its standardization, dependability, and integrity [1]. To date, ChatGPT passed all 3 exams on the United States Medical Licensing Examination [4,5]. The use of ChatGPT to enhance the development of a self-evaluation tool for assessing implicit bias in medical students showed promising consistency with student ratings, particularly for non-stereotypical statements, suggesting potential for its application in medical education and the evaluation of ethnic stereotypes [6]. In parasitology, ChatGPT’s performance is not yet comparable to those of medical students [7]. ChatGPT demonstrated a good understanding of the principles of statistics [8], but also it was found that it can make fatal errors with simple calculations [9].

### Objectives

In this study, we assess the performance of GPT-3.5 and GPT-4 as study tools in solving biostatistical problems and locating any potential drawbacks or issues that might arise from using ChatGPT in medical education, particularly in solving practical biostatistical problems.

## Methods

### Ethics statement

This study was conducted without human participants; therefore, the study did not require an institutional ethics review.

### Study design

In this descriptive study, GPT-3.5 and GPT-4 were tested to solve biostatistical problems.

### Setting

Biostatistical problems were selected from the “Oxford handbook of medical statistics” by Peacock and Peacock [10]. Some questions tabulated in the handbook are transformed into textual questions to ChatGPT. Ten biostatistical problems were randomly chosen and used as text-based input for conversation with ChatGPT, particularly with GPT-3.5. If ChatGPT did not solve a problem in the first attempt, we used different modifications and fed the application additional details to solve the problem correctly. We used the current versions of GPT-3.5 and GPT-4. The conversations with GPT-3.5 and GPT-4 are provided as supplementary materials.

### Variables

The variables were ChatGPT’s answers to 10 biostatistical problems.

### Data sources and measurement

The data are from ChatGPT’s answers. The correctness of answers was assessed as “yes” or “no.”

### Bias

There was no bias in the script description for solving 10 biostatistical problems because the researchers did the work.

### Study size

There was no need to calculate the study size. Only ChatGPT was targeted for the study.

### Statistical methods

Descriptive statistics were used to analyze the answers by ChatGPT.

## Results

### Main results

The performance of GPT-3.5 and GPT-4 in different domains in biostatistics is compared in Table 1. The complete conversations with GPT-3.5 and GPT-4 are provided as supplementary material (Supplements 1 and 2, respectively). GPT-3.5 successfully solved the following 5 practical problems: categorical data, cross-sectional study, measuring reliability, probability properties, and the t-test for paired data. It correctly solved the tasks for the confidence interval and binomial distribution in the third attempt. Statistical problems related to the chi-square test, one-way analysis of variance (ANOVA), and sample size were not correctly solved in 3 attempts. Compared to GPT-3.5, GPT-4 additionally solved the confidence interval task in the first attempt, the binomial distribution and chi-square test tasks in the second attempt, and the sample size calculation and one-way ANOVA in the third attempt.

The first question was about the 95% confidence interval. In the first attempt, GPT-3.5 incorrectly calculated the standard error. The calculation was closer to the correct result in the second attempt but still wrong. In the third attempt, ChatGPT successfully calculated the 95% confidence interval. The result was slightly different because of altered rounding in the GPT-4 version, which provided the correct response on the first attempt.

The second biostatistical problem was related to the probability of a binomial distribution. In the third attempt, after the task was broken down into 2 simpler parts, GPT-3.5 succeeded in solving the problem. GPT-4 did not give the correct final answer on the first attempt, but when we provided the correct final result, this version found the right formula and gave the correct result, but the 3.5 version failed. The following 2 questions related to categorical data and cross-sectional study were done correctly by both versions in the first attempt.

The question concerning the chi-square test was not solved in 3 attempts by GPT-3.5. In the first attempt, the chatbot failed to create the contingency table correctly. In the next try, it was unable to sum up the total number of patients. Finally, in the third attempt, it failed to calculate the correct chi-square test value. GPT-4 created the right contingency table and gave the correct total number of patients, but made mistakes in calculating the expected frequencies on the first try. After we provided the correct expected frequencies, ChatGPT correctly gave the chi-square test value in the second attempt.

In solving the task related to one-way ANOVA, GPT-3.5 failed to calculate the mean value for the first and third groups in the first attempt. In the next attempt, the formula for the sum of squares within groups (SSW) was wrong. After 3 attempts, the correct answer was offered to GPT-3.5, but ChatGPT could not develop the right solutions. GPT-4 failed to give the proper final result in the first attempt. The task was broken down into 2 simple parts. First, the overall mean was corrected; second, within-group variation (SSW) was fixed in all 3 groups. Finally, GPT-4 offered the correct result for between-group variation (SSB) and the correct F-value in the third attempt. GPT-3.5 provided inaccurate answers to question 9 in 3 attempts related to sample size calculation. Firstly, it miscalculated the sample size and got the wrong results in the subsequent 2 attempts. GPT-4 failed to give the correct final result on the first attempt. The sample size was miscalculated on the second attempt after the correct answer was offered to GPT-4 again. Finally, on the third attempt, when we provided the right formula, the chatbot gave the correct answer. Both versions accurately answered the last question related to the t-test for paired data.

## Discussion

### Key results

The assessment of ChatGPT accuracy in the 10 biostatistical problems revealed that GPT-3.5 and GPT-4 showed below-average performance, with a response rate of 5 out of 10 and 6 out of 10 in the first attempt, respectively. GPT-3.5 exhibited average performance within the first 3 attempts, with 7 out of 10 responses, and GPT-4 provided all correct responses within the first 3 attempts (when we grade tests, a score of 7 out of 10 is usually graded as an average performance).

### Interpretation

From the subject-matter perspective, GPT-3.5 failed to correctly solve 3 commonly used statistical analyses, which are usually compulsory in an introductory course syllabus, indicating that students must be aware that this tool, even when providing and calculating different statistical analyses, can be wrong. The newer version, GPT-4, will likely offer better responses. However, our findings indicate that GPT-4 must be precisely guided and monitored during the calculations. ChatGPT provided answers in a logically clear and organized manner. Additionally, chatbot-generated responses were long and detailed. However, ChatGPT did not refuse to answer the question due to a lack of knowledge. It claimed its result was correct, but eventually accepted suggestions and hints.

### Comparison with previous studies

These findings echo and confirm results from a recent publication emphasizing that students still must acquire subject foundations, maintain and develop critical thinking, and not unquestioningly trust chatbot-generated responses [11]. In addition, easy natural language conversation with ChatGPT requires acquiring effective techniques for generating prompts and evaluating responses. Students also expressed concerns about the accuracy of ChatGPT, and they were aware that model improvement is needed [11]. Furthermore, students demonstrated great interest, admiration, and motivation for learning and applying ChatGPT in that study. Previous research also showed that ChatGPT should be supported by human judgment due to a lack of critical thinking, generation, accuracy, and critical appraisal [9].

Statistical knowledge is the foundation for developing skills needed for evaluating evidence critically, complex clinical practice decision-making, and, overall, applying evidence-based medicine [12]. Therefore, the potential benefits of ChatGPT as an educational tool require investigation in biostatistics. The current study assessed ChatGPT’s performance, particularly GPT-3.5, as lower than the previous estimated performance based on questions on statistics principles [8]. One out of 15 questions in that study remained incorrectly answered after several attempts. Notably, this question was also related to problems in calculating the mean value of the dataset. In our study, we noticed that in questions with many complex mathematical operations, such as one-way ANOVA, the chi-square test, and sample size calculation, ChatGPT chose wrong formulas and made frequent calculation mistakes. Even GPT-4 had to be carefully guided to calculate one-way ANOVA. Dao and Le [9], who investigated the effectiveness of ChatGPT in mathematical problem-solving, showed that ChatGPT did well on some levels while doing poorly on others. At the least complex level, 83% of the questions were answered correctly by ChatGPT; however, as the difficulty of the questions increased, the accuracy rate fell to 10% at the most advanced level. Even though language models like ChatGPT have made tremendous strides recently, they still struggle to handle graphical data, understand complex mathematical concepts, and solve challenging mathematical problems [9]. Text-based inputs might significantly affect statistical analysis, which extensively relies on diagrams, tables, and graphs.

GPT 3.5 cannot use graphical forms of data reporting, which require additional effort to be explained in text form for a chatbot to understand. If a question is ambiguous or incomprehensible, it is recommended to rephrase it until ChatGPT gets a clear and precise question [13]. Recently, distinctive patterns of ChatGPT have been recognized [14], which seem to have generally been confirmed through ChatGPT’s responses in our study.

### Limitations

This study has several limitations. First, number of biostatistical problems was small, and additional extensive research is needed. Second, the performance of ChatGPT was evaluated based on the number of correct responses without considering the demand level. There were no pre-established guidelines for asking questions and leading conversations with ChatGPT. Our assistance and questions were adjusted to the statistical problem. Additionally, tabulated data had to be transformed into text-based inputs for GPT-3.5.

### Suggestions for further studies

This study demonstrated that ChatGPT’s algorithm has improved. However, incorrect ChatGPT answers might be generated if the questions are ambiguous, unclear, out-of-domain, biased, or trained by inaccurate data [15]. Therefore, further research must be initiated to create guidelines and recommendations on how to design text inputs. Secondly, further studies must evaluate proposed ChatGPT training programs for students and follow improvements in AI algorithms.

### Conclusion

The present study provided evidence about the performance of GPT-3.5 and GPT-4 in solving biostatistical problems. In the first 3 attempts, GPT-3.5 showed an average level of performance, while GPT-4 exhibited good performance. Consequently, medical students should be aware of ChatGPT’s limitations and be careful when incorporating this model into medical education. Therefore, professors should introduce all characteristics of this platform to students, specifying its advantages and pointing out its disadvantages. Academic societies should encourage the implementation of ChatGPT in medical education, while in parallel extensively testing the model to protect against overlooking its limitations. Eventually, testing by itself will make ChatGPT even better.

## Figures and Tables

**Figure f1-jeehp-20-28:**
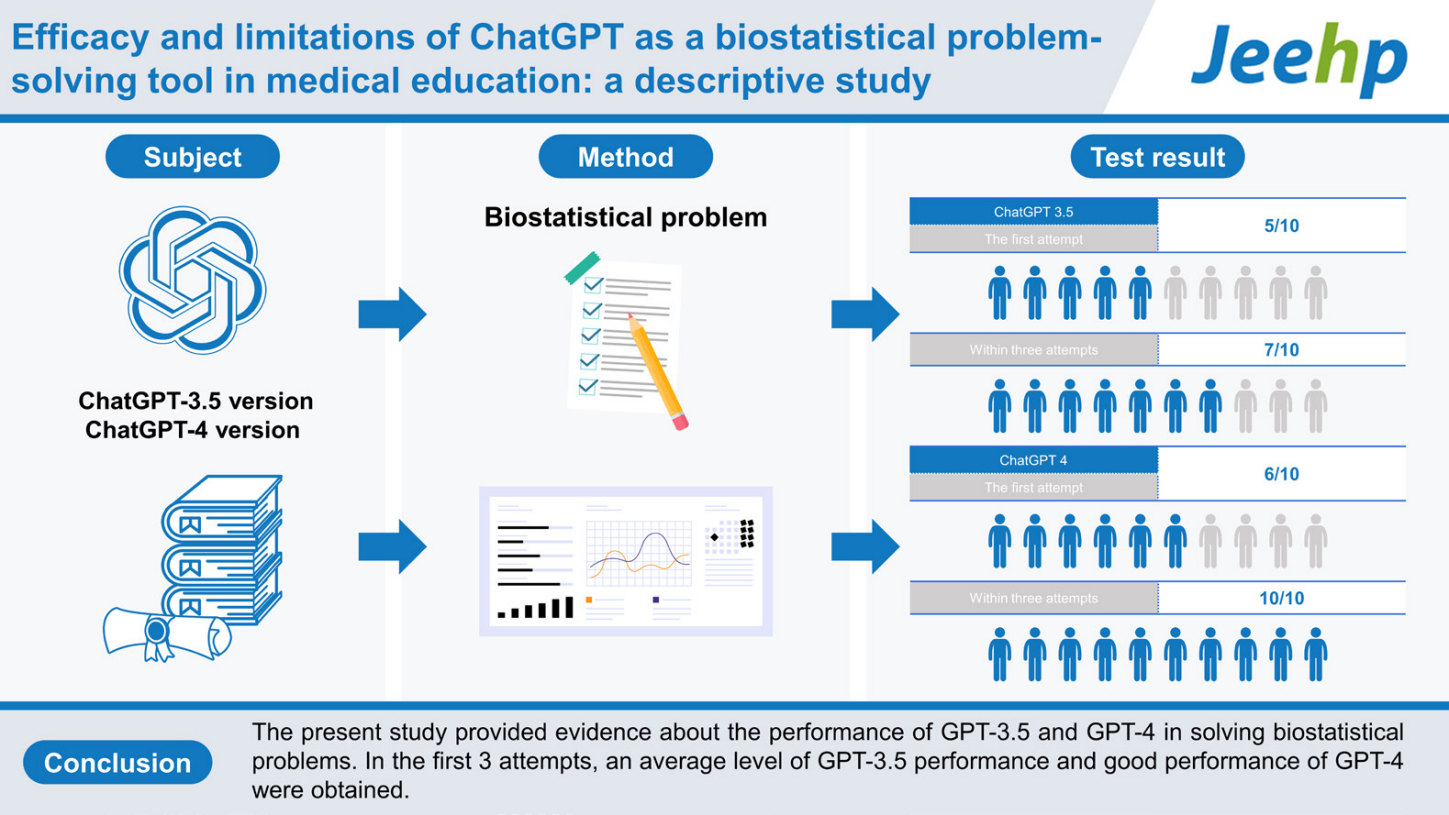


**Table 1.** Comparative analysis between GPT-3.5 and GPT-4 for certain domains in biostatistics

Domain	Version of ChatGPT	Correct in the first attempt	Correct in the second attempt	Correct in the third attempt	Incorrect after more than 3 attempts
95% Confidence interval	V 3.5			Y	
	V 4	Y			
Binomial distribution	V 3.5			Y	
	V 4		Y		
Categorical data	V 3.5	Y			
	V 4	Y			
Cross-sectional study	V 3.5	Y			
	V 4	Y			
The chi-square test	V 3.5				Y
	V 4		Y		
Measuring reliability	V 3.5	Y			
	V 4	Y			
One-way analysis of variance	V 3.5				Y
	V 4			Y	
Probability: properties	V 3.5	Y			
	V 4	Y			
Sample size calculation	V 3.5				Y
	V 4			Y	
T-test for paired data	V 3.5	Y			
	V 4	Y			

Y, yes.

## Supplementary materials

Supplement 1. GPT-3.5’s answers to 10 questions on biostatics.

Supplement 2. GPT-4’s answers 10 questions on biostatics.

Supplement 3. Audio recording of the abstract.
