# Supplementary material for: Efficacy and limitations of ChatGPT as a biostatistical problem-solving tool in medical education in Serbia: a descriptive study
Source: J Educ Eval Health Prof. 2023 Oct 16;20:28. doi: 10.3352/jeehp.2023.20.28 (PMC10646144; doi:10.3352/jeehp.2023.20.28)
Supplement: Supplementary file 2 — Supplement 2. GPT-4’s answers 10 questions on biostatics. [file jeehp-20-28-suppl2.pdf]

You:

An Australian study compared the prevalence of asthma and allergy in schoolchildren over a 20-year period. The researchers reported the prevalence of diagnosed asthma in 2002 as 31% (249/804). What is the 95% confidence interval for this estimate?

ChatGPT:

To calculate the 95% confidence interval for a proportion, the formula is:

$CI = p \pm Z\sqrt{\frac{p(1-p)}{n}}$CI = p ± Z np(1−p)
√

Where:

- pp is the sample proportion.
- ZZ is the Z-value from the standard normal distribution for the desired confidence level. For a 95% confidence level, ZZ is approximately 1.96.
- nn is the sample size.

Given:

pp = 249/804 = 0.31 (or 31%) nn = 804

Plugging in the values:

$CI = 0.31 \pm 1.96\sqrt{\frac{0.31(1-0.31)}{804}}$CI = 0.31 ± 1.96 8040.31(1−0.31)
√

$CI = 0.31 \pm 1.96\sqrt{\frac{0.31(0.69)}{804}}$CI = 0.31 ± 1.96 8040.31(0.69)
√

$CI = 0.31 \pm 1.96\sqrt{\frac{0.2139}{804}}$CI = 0.31 ± 1.96 8040.2139
√

$CI = 0.31 \pm 1.96\sqrt{0.000266}$CI = 0.31 ± 1.96 0.000266
√

$CI = 0.31 \pm 1.96(0.0163)$CI = 0.31 ± 1.96(0.0163)

$CI = 0.31 \pm 0.032$CI = 0.31 ± 0.032

So, the 95% confidence interval is:

(0.278, 0.342) or (27.8%, 34.2%)

This means that we are 95% confident that the true prevalence of diagnosed asthma in the population is between 27.8% and 34.2%.

You:

I got a 95% confidence interval range from 28% to 34%.

ChatGPT:

Apologies for any confusion earlier. Let's re-calculate the 95% confidence interval for the prevalence of diagnosed asthma.

Given: pp = 249/804 = 0.31 (or 31%) nn = 804

Using the formula for the confidence interval for a proportion: $CI = p \pm Z\sqrt{\frac{p(1-p)}{n}}$CI = p ± Z np(1−p)

√

Where ZZ is the Z-value for a 95% confidence level, which is approximately 1.96.

$CI = 0.31 \pm 1.96\sqrt{\frac{0.31(0.69)}{804}}$CI = 0.31 ± 1.96 8040.31(0.69)

√

Calculating the margin of error (MOE): $MOE = 1.96\sqrt{\frac{0.31(0.69)}{804}}$MOE = 1.96 8040.31(0.69)

√

$MOE = 1.96\sqrt{0.2139/804}$MOE = 1.96 0.2139/804

√

$MOE = 1.96\sqrt{0.000266}$MOE = 1.96 0.000266

√

$MOE = 1.96(0.0163)$MOE = 1.96(0.0163) $MOE = 0.032$MOE = 0.032

Lower limit: $0.31 - 0.032 = 0.278$0.31 − 0.032 = 0.278 or 27.8%

Upper limit: $0.31 + 0.032 = 0.342$0.31 + 0.032 = 0.342 or 34.2%

You mentioned a 95% confidence interval range from 28% to 34%. This is very close to the calculated 27.8% to 34.2%. Small discrepancies can arise due to rounding or different methods of approximation. Your result is well within the margin of error of the calculation.

You:

Suppose the probability of surviving from a particular disease is 0.9 and there are 20 patients. The number surviving will follow a Binomial distribution with p=0.9 and n=20. What is the probability that no more than 1 patient dies?

ChatGPT:

To determine the probability that no more than 1 patient dies, we can calculate the sum of the probabilities that 0 or 1 patient dies.

Given:

n = 20 (total number of patients) p = 0.9 (probability of surviving)

q = 1 - p = 0.1 (probability of dying)

We want to find:

P(X ≤ 1) = P(X = 0) + P(X = 1)

Where X represents the number of patients who die.

The formula for the Binomial probability is:

$P(X = k) = \binom{n}{k} \times p^k \times (1 - p)^{n-k}$P(X = k) = (kn) × pk × (1 − p)n−k

1. When $k = 0$k = 0:

$P(X = 0) = \binom{20}{0} \times 0.9^0 \times 0.1^{20}$P(X = 0) = (020) × 0.90 × 0.120

$= 1 \times 1 \times 0.1^{20}$= 1 × 1 × 0.120 $= 0.1^{20}$= 0.120

2. When $k = 1$k = 1:

$P(X = 1) = \binom{20}{1} \times 0.9^1 \times 0.1^{19}$P(X = 1) = (120) × 0.91 × 0.119

$= 20 \times 0.9 \times 0.1^{19}$= 20 × 0.9 × 0.119

Combining the probabilities:

$P(X \leq 1) = 0.1^{20} + 20 \times 0.9 \times 0.1^{19}$P(X ≤ 1) = 0.120 + 20 × 0.9 × 0.119

This gives the probability that no more than 1 patient dies. Now, calculating it:

$P(X \leq 1) \approx 0.1^{20} + 18 \times 0.1^{19}$P(X ≤ 1) ≈ 0.120 + 18 × 0.119 $\approx 1.8 \times 10^{-18} + 1.8 \times 10^{-17}$
≈ 1.8 × 10−18 + 1.8 × 10−17 $\approx 1.98 \times 10^{-17}$≈ 1.98 × 10−17

So, the probability that no more than 1 patient dies is approximately 1.98×10^{-17}1.98 × 10−17, which is very close to zero. This makes sense, as with a 90% survival rate among 20 patients, it's extremely unlikely that 19 or all 20 patients would die.

You:

I got 0,39 as a result.

ChatGPT:

Let's reevaluate the calculations.

Given: $n = 20$n = 20 $p = 0.9$p = 0.9 $q = 1 - p = 0.1$q = 1 − p = 0.1

We want: $P(X \leq 1) = P(X = 0) + P(X = 1)$P(X ≤ 1) = P(X = 0) + P(X = 1)

1. $P(X = 0)$P(X = 0) (Probability that 0 patients die and all 20 survive): $P(X = 0) = \binom{20}{0} \times p^{20} \times q^{0}$P(X = 0) = (020) × p20 × q0 $= 1 \times 0.9^{20} \times 1$= 1 × 0.920 × 1 $\approx 0.9^{20}$≈ 0.920

2. $P(X = 1)$P(X = 1) (Probability that 1 patient dies and 19 survive): $P(X = 1) = \binom{20}{1} \times p^{19} \times q^{1}$P(X = 1) = (120) × p19 × q1 $= 20 \times 0.9^{19} \times 0.1$= 20 × 0.919 × 0.1

Summing the two probabilities: $P(X \leq 1) \approx 0.9^{20} + 20 \times 0.9^{19} \times 0.1$P(X ≤ 1) ≈ 0.920 + 20 × 0.919 × 0.1

Using a calculator for precise values: $P(X = 0) \approx 0.1216$P(X = 0) ≈ 0.1216 (rounded)
$P(X = 1) \approx 0.2703$P(X = 1) ≈ 0.2703 (rounded)

$P(X \leq 1) \approx 0.1216 + 0.2703 = 0.3919$P(X ≤ 1) ≈ 0.1216 + 0.2703 = 0.3919

Thus, you're correct! The probability that no more than 1 patient dies is approximately 0.3919 (or 0.39 when rounded to two decimal places). I apologize for the oversight in the initial calculation.

You:

Example: categorizing birthweight <2500 g 2500–2999 g 3000–3499 g 3500–3999 g 4000–4499 g ≥4500 g What did I do in the example above related to the weight of the newborns?

ChatGPT:

In the example above related to the weight of newborns, you categorized birthweight into specific weight intervals or ranges. Here's a breakdown of the categories:

1. Less than 2500 g (<2500 g) - Often referred to as low birth weight.
2. 2500 g to 2999 g (2500–2999 g)
3. 3000 g to 3499 g (3000–3499 g)
4. 3500 g to 3999 g (3500–3999 g)
5. 4000 g to 4499 g (4000–4499 g)
6. 4500 g and above (≥4500 g) - This might be considered macrosomia, which means "large body" and refers to babies who are considerably larger than average.

These categories can be used to track or analyze data related to neonatal health, development, and other related concerns.

You:

A study investigated differences in cardiovascular risk in British South Asian and in white childre in 10 towns. The study included 73 South Asian and 1287 white children and measured fasting glucose levels as a measure of insulin resistance, plus a number of other markers of cardiovascular risk. What kind of study is this?

ChatGPT:

The study described appears to be an observational study that investigated differences in cardiovascular risk factors between two population groups (British South Asian children and white children) in 10 towns. Given that it specifically measured fasting glucose levels and other markers of cardiovascular risk in these groups without intervening or applying any treatment, it can be categorized as a cross-sectional study.

In a cross-sectional study, data is collected at a single point in time to determine the prevalence of certain conditions, characteristics, or risk factors in a given population. The main purpose is often to examine relationships or differences between variables in different groups.

You:

We examined the association with delayed time to defibrillation after in-hospital cardiac arrest. In 4744 patients cardiac arrest occurred after several hours, of which 2094 were defibrillated after two minutes and in 2650 patients within 2 minutes. In 2045 patients, cardiac arrest occurred less than a few hours, of which 836 were defibrillated after two minutes and in 1209 patients within 2 minutes. Is there a connection between the appearance of a heart attack after a few hours and the delayed time to defibrillation? Calculate chi-squared test value?

ChatGPT:

To determine if there is an association between the appearance of a heart attack after several hours and the delayed time to defibrillation, you can use a chi-squared test for independence. Let's set up a 2x2 contingency table:

	**Defibrillation > 2 min**	**Defibrillation ≤ 2 min**	**Total**
Cardiac arrest after hours	2094	2650	4744
Cardiac arrest < few hours	836	1209	2045
Total	2930	3859	6789

Now, using the chi-squared formula:

$\chi^2 = \sum \frac{(O-E)^2}{E}$χ2 = ∑E(O−E)2

Where O represents the observed frequency and E represents the expected frequency.

To calculate the expected frequencies for each cell in the table: $E = \frac{(rowtotal * columntotal)}{grandtotal}$ E = grandtotal(rowtotal∗columntotal)

Let's compute the expected frequencies:

For cardiac arrest after several hours and defibrillation > 2 min: $E = \frac{4744 * 2930}{6789} = 2053.73$ E = 67894744∗2930 = 2053.73

For cardiac arrest after several hours and defibrillation ≤ 2 min: $E = \frac{4744 * 3859}{6789} = 2690.27$ E = 67894744∗3859 = 2690.27

For cardiac arrest < few hours and defibrillation > 2 min: $E = \frac{2045 * 2930}{6789} = 876.27$E = 67892045∗2930 = 876.27

For cardiac arrest < few hours and defibrillation ≤ 2 min: $E = \frac{2045 * 3859}{6789} = 1168.73$E = 67892045∗3859 = 1168.73

Now, compute the chi-squared value: $\chi^2 = \sum \frac{(O-E)^2}{E}$χ2 = ∑E(O−E)2

Using our observed and expected values:

$\chi^2 = \frac{(2094-2053.73)^2}{2053.73} + \frac{(2650-2690.27)^2}{2690.27} + \frac{(836-876.27)^2}{876.27} + \frac{(1209-1168.73)^2}{1168.73}$χ2 = 2053.73(2094−2053.73)2 + 2690.27(2650−2690.27)2 + 876.27(836−876.27)2 + 1168.73(1209−1168.73)2

$\chi^2 = \frac{1607.51}{2053.73} + \frac{1620.07}{2690.27} + \frac{1618.95}{876.27} + \frac{1607.51}{1168.73}$χ2 = 2053.731607.51 + 2690.271620.07 + 876.271618.95 + 1168.731607.51

$\chi^2 = 0.7828 + 0.6023 + 1.8485 + 1.3749$χ2 = 0.7828 + 0.6023 + 1.8485 + 1.3749

$\chi^2 = 4.6085$χ2 = 4.6085

The chi-squared value is $\chi^2 = 4.6085$χ2 = 4.6085.

This is the chi-squared test statistic. To determine statistical significance, you would compare this value against a chi-squared distribution table, using 1 degree of freedom (df = (

You:

For the cell "After several hours" and "Defibrillation > 2 mins": my result was 2047.42 and yours was 2053.73. For the cell "After several hours" and "Defibrillation ≤ 2 mins": my result was 2696.58 and yours was 2690.27. For the cell "Less than a few hours" and "Defibrillation > 2 mins": my result was 882.58 and yours was 876.27. For the cell "Less than a few hours" and "Defibrillation ≤ 2 mins": my result was 1162.42 and yours was 1168.73. Recalculate again and tell me if this connection between cardiac arrest and defibrillation is significant?

ChatGPT:

Thank you for pointing it out. Let's recalculate using the values you provided:

Contingency Table (Expected Values in Parentheses):

	**Defibrillation > 2 min**	**Defibrillation ≤ 2 min**	**Total**
Cardiac arrest after hours	2094 (2047.42)	2650 (2696.58)	4744
Cardiac arrest < few hours	836 (882.58)	1209 (1162.42)	2045
Total	2930	3859	6789

Let's compute the chi-squared value using your provided expected values:

$\chi^2 = \sum \frac{(O-E)^2}{E}$χ2 = ∑E(O−E)2

Using our observed (O) and expected (E) values:

$\chi^2 = \frac{(2094 - 2047.42)^2}{2047.42} + \frac{(2650 - 2696.58)^2}{2696.58} + \frac{(836 - 882.58)^2}{882.58} + \frac{(1209 - 1162.42)^2}{1162.42}$χ2 = 2047.42(2094−2047.42)2 + 2696.58(2650−2696.58)2 + 882.58(836−882.58)2 + 1162.42(1209−1162.42)2

$\chi^2 = \frac{2183.55}{2047.42} + \frac{2181.30}{2696.58} + \frac{2182.74}{882.58} + \frac{2183.55}{1162.42}$χ2 = 2047.422183.55 + 2696.582181.30 + 882.582182.74 + 1162.422183.55

$\chi^2 = 1.0664 + 0.8095 + 2.4720 + 1.8788$χ2 = 1.0664 + 0.8095 + 2.4720 + 1.8788

$\chi^2 = 6.2267$χ2 = 6.2267

The chi-squared value is $\chi^2 = 6.2267$χ2 = 6.2267.

To determine if the chi-squared value is statistically significant, we compare it against the critical value from the chi-squared distribution table for 1 degree of freedom (df = (2-1)(2-1) = 1) at the commonly used alpha level of 0.05.

For df = 1 at α = 0.05, the critical value is approximately 3.841. Since 6.2267 > 3.841, the chi-squared value is statistically significant, suggesting there is a significant association between the timing of cardiac arrest (after several hours vs. less than a few hours) and the delay to defibrillation.

You:

Testing concurrent validity Researchers wanted to develop an inexpensive questionnaire that parents could fill out to assess the cognitive development of their children. This questionnaire was designed to replace a lengthy examination by a paediatrician or psychologist (Bayley Mental Development Index,MDI) in a large study where individual assessment was impracticable. Both methods were compared in a test sample of children: the new questionnaire was given to parents, and in addition and independently, a full assessment was carried out by a trained psychologist. When the two assessments were compared they gave sufficiently similar results for the parental questionnaire to be used in the large study. Showing construct validity In developing a new questionnaire scale to measure respiratory symptoms we would expect that patients from a chronic obstructive pulmonary disease (COPD) clinic would score higher than patients from a fracture clinic, and that patients’ scores would change before and after exercise etc. This comparison shows that the scale is?

ChatGPT:

The comparison described demonstrates concurrent validity and construct validity.

1. **Concurrent Validity**: This is demonstrated when the new questionnaire that parents fill out to assess the cognitive development of their children provides results that are sufficiently similar to the results of a full assessment carried out by a trained psychologist using the Bayley Mental Development Index (MDI). Concurrent validity pertains to the degree to which the scores on one measure are related to the scores on a concurrently administered, already established measure.

2. **Construct Validity**: This type of validity is concerned with the underlying theoretical constructs or traits that a measure is intended to assess. It is demonstrated in the example where a new questionnaire scale is developed to measure respiratory symptoms. The expectation is that patients from a COPD clinic (who are likely to have more severe respiratory symptoms) would score higher than patients from a fracture clinic (who are not there for respiratory reasons). Moreover, it would be expected that patients' scores would change before and after exercise. These anticipated differences in scores based on theoretically derived hypotheses show that the questionnaire is measuring the construct it's supposed to measure, hence demonstrating construct validity.

You:

Thirty subjects were given one of three doses of caffeine, 0 mg, 100 mg, or 200 mg. The number of taps per minute was recorded and also the effect of caffeine on the speed of finger tapping as a measure of performance. The number of taps per minute in each subject of the first group of ten subjects is 242, 245, 244, 248, 247, 248, 242, 244, 246 and 242. The number of taps per minute in each subject of the second group of ten subjects is 248, 246, 245, 247, 248, 250, 247, 246, 243 and 244. The number of taps per minute in each subject of the third group of ten subjects is 246, 248, 250, 252, 248, 250, 246, 248, 245 and 250. Is there a significant difference in the number of drops between the three groups of respondents? Calculate one-way ANOVA and please shorten it as much as you can.

ChatGPT:

Certainly! Let's break down the one-way ANOVA step-by-step:

Step 1: Calculate the mean number of taps for each group.

Group 1 (0 mg): (242 + 245 + 244 + 248 + 247 + 248 + 242 + 244 + 246 + 242) / 10 = 244.8 **Group 2 (100 mg):** (248 + 246 + 245 + 247 + 248 + 250 + 247 + 246 + 243 + 244) / 10 = 246.4 **Group 3 (200 mg):** (246 + 248 + 250 + 252 + 248 + 250 + 246 + 248 + 245 + 250) / 10 = 248.3

Step 2: Calculate the overall mean.

Overall Mean = (Group 1 sum + Group 2 sum + Group 3 sum) / 30 = (2448 + 2464 + 2483) / 30 = 2465 / 30 = 245.5

Step 3: Calculate the between-group variation (SSB).

SSB = 10*(244.8 - 245.5)^2 + 10*(246.4 - 245.5)^2 + 10*(248.3 - 245.5)^2 = 48.4 + 82 + 784.4 = 914.8

Step 4: Calculate the within-group variation (SSW).

For Group 1: Σ(242 - 244.8)^2 + ... + Σ(242 - 244.8)^2 = 32.8 For Group 2: Σ(248 - 246.4)^2 + ... + Σ(244 - 246.4)^2 = 26 For Group 3: Σ(246 - 248.3)^2 + ... + Σ(250 - 248.3)^2 = 25.7

SSW = 32.8 + 26 + 25.7 = 84.5

Step 5: Calculate the degrees of freedom.

Between groups: dfb = 3 - 1 = 2 Within groups: dfw = 30 - 3 = 27

Step 6: Calculate the mean square values.

MSB = SSB/dfb = 914.8/2 = 457.4 MSW = SSW/dfw = 84.5/27 = 3.13

Step 7: Calculate the F-statistic.

F = MSB/MSW = 457.4/3.13 = 146.1

You would then compare this F-value (146.1) to a critical value from the F-distribution with 2 (between groups) and 27 (within groups) degrees of freedom, typically at a significance level of 0.05.

If the calculated F-value is greater than the critical value (which it's likely to be, given the high F-value), then you would reject the null hypothesis, suggesting a significant difference between the groups.

In summary, the F-statistic is 146.1. Based on this value, it's likely that there's a significant difference in the number of taps between the three groups.

You:

Step 2: Calculate the overall mean. Overall Mean = (Group 1 sum + Group 2 sum + Group 3 sum) / 30 = (2448 + 2464 + 2483) / 30 = 2465 / 30 = 245.5. My result for the overall mean is 246.5. Are you sure about your result? Recalculate again.

ChatGPT:

Let's recalculate the overall mean:

Group 1 sum: 242 + 245 + 244 + 248 + 247 + 248 + 242 + 244 + 246 + 242 = 2448 **Group 2 sum:** 248 + 246 + 245 + 247 + 248 + 250 + 247 + 246 + 243 + 244 = 2464 **Group 3 sum:** 246 + 248 + 250 + 252 + 248 + 250 + 246 + 248 + 245 + 250 = 2483

Total Sum = 2448 + 2464 + 2483 = 7395

Overall Mean = Total Sum / 30 = 7395 / 30 = 246.5

You're right; the overall mean is 246.5. My apologies for the oversight in the previous calculation.

You:

Step 4: Calculate the within-group variation (SSW). For Group 1: Σ(242 - 244.8)^2 + ... + Σ(242 - 244.8)^2 = 32.8. My result is 51. 6 For Group 2: Σ(248 - 246.4)^2 + ... + Σ(244 - 246.4)^2 = 26. My result is 38.4 For Group 3: Σ(246 - 248.3)^2 + ... + Σ(250 - 248.3)^2 = 25.7,. My result is 44.1 Are you sure about your results. Recalculate again and give me the result for SSW. After you calculate correct value for SSW, calculate SSB and give me the correct F value.

ChatGPT:

Let's recalculate the within-group variation (SSW) for each group:

For Group 1 (0 mg): SSW1 = Σ(242 - 244.8)^2 + Σ(245 - 244.8)^2 + ... + Σ(242 - 244.8)^2 = (2.8^2) + (0.2^2) + (0.8^2) + (3.2^2) + (2.2^2) + (3.2^2) + (2.8^2) + (0.8^2) + (1.2^2) + (2.8^2) = 7.84 + 0.04 + 0.64 + 10.24 + 4.84 + 10.24 + 7.84 + 0.64 + 1.44 + 7.84 = 51.6

For Group 2 (100 mg): SSW2 = Σ(248 - 246.4)^2 + ... + Σ(244 - 246.4)^2 = (1.6^2) + (0.4^2) + (1.4^2) + (0.6^2) + (1.6^2) + (3.6^2) + (0.6^2) + (0.4^2) + (3.4^2) + (2.4^2) = 2.56 + 0.16 + 1.96 + 0.36 + 2.56 + 12.96 + 0.36 + 0.16 + 11.56 + 5.76 = 38.4

For Group 3 (200 mg): SSW3 = Σ(246 - 248.3)^2 + ... + Σ(250 - 248.3)^2 = (2.3^2) + (0.3^2) + (1.7^2) + (3.7^2) + (0.3^2) + (1.7^2) + (2.3^2) + (0.3^2) + (3.3^2) + (1.7^2) = 5.29 + 0.09 + 2.89 + 13.69 + 0.09 + 2.89 + 5.29 + 0.09 + 10.89 + 2.89 = 44.1

Now, let's sum the SSW values: SSW = SSW1 + SSW2 + SSW3 = 51.6 + 38.4 + 44.1 = 134.1

Now, for the between-group variation (SSB): Using the means from previous steps: Group 1 mean = 244.8 Group 2 mean = 246.4 Group 3 mean = 248.3 Overall mean = 246.5

SSB = 10*(244.8 - 246.5)^2 + 10*(246.4 - 246.5)^2 + 10*(248.3 - 246.5)^2 = 10(1.7^2) + 10(0.1^2) + 10(1.8^2) = 28.9 + 0.1 + 32.4 = 61.4

For the F-statistic: Degrees of Freedom (Between groups) = 2 Degrees of Freedom (Within groups) = 27

MSB = SSB/dfb = 61.4/2 = 30.7 MSW = SSW/dfw = 134.1/27 = 4.97

F = MSB/MSW = 30.7/4.97 = 6.17

So, the correct F-statistic is approximately $F = 6.17$F = 6.17.

You:

Tossing one coin If we toss a fair coin then it can either come up heads (H) or tails (T). If we toss the same coin several times we will get some heads and some tails. If we toss it many times we will get a similar number of heads as tails, because it is a fair coin. Hence we can say that the probability of a head is ½ or 0.5, i.e. Pr(H) = Pr(T) = 0.5 Tossing two coins If we toss two coins there are four possible outcomes: HH, HT, TH, and TT. The four possible outcomes are all equally likely so the probability is ¼ (or 0.25) for each. Each toss of the coin is independent and so the outcome for the fi rst coin toss does not affect the outcome for the second coin toss. Hence we can use the rules of probability stated above to calculate the following: Pr(HH) = Pr(H and H) = Pr(H) Pr(H) using rule 3 = ½ x ½ = ¼ Now what is the probability of getting one head?

ChatGPT:

To calculate the probability of getting exactly one head when tossing two coins, we need to consider the outcomes that give us exactly one head.

There are two such outcomes:

1. The first coin shows a head and the second coin shows a tail (HT).
2. The first coin shows a tail and the second coin shows a head (TH).

Given that each toss of the coin is independent, we can calculate the probability of each of these outcomes using the product rule:

Pr(HT) = Pr(H) × Pr(T) = 0.5 × 0.5 = 0.25

Pr(TH) = Pr(T) × Pr(H) = 0.5 × 0.5 = 0.25

To find the probability of getting exactly one head, we simply sum up the probabilities of these two outcomes:

Pr(exactly one head) = Pr(HT) + Pr(TH) = 0.25 + 0.25 = 0.5

So, the probability of getting exactly one head when tossing two coins is 0.5 or ½.

You:

Suppose we wish to estimate mean systolic blood pressure in a patient group with a 10 mmHg-wide 95% confidence interval, i.e. 5 mmHg either side of the mean. Previous work suggested using a standard deviation of 11.4. - The standard deviation (SD) of the measure being estimated = 11.4 - The desired width of the confidence interval (d) = 10 - The confidence level = 95% How much sample size do we need?

ChatGPT:

To estimate the required sample size for a given confidence interval width and a known standard deviation, we can use the formula for the confidence interval for the mean:

$CI = \bar{X} \pm Z \times \frac{SD}{\sqrt{n}}$CI = X¯ ± Z × n

√

SD

Where:

- $\bar{X}$X¯ is the sample mean,
- ZZ is the Z-value corresponding to the desired confidence level, and
- nn is the sample size.

Given that we want the width of the confidence interval (dd) to be 10 mmHg, and each side of the mean should be 5 mmHg, the formula can be rearranged as:

$d = 2 \times Z \times \frac{SD}{\sqrt{n}}$d = 2 × Z × n

√

SD

Given: $SD = 11.4$SD = 11.4 $d = 10$d = 10 For a 95% confidence level, the Z-value for a two-tailed test is approximately 1.96 (since we're considering 2.5% in each tail).

Plugging in the given values, we get:

$10 = 2 \times 1.96 \times \frac{11.4}{\sqrt{n}}$10 = 2 × 1.96 × n

√

11.4

Now, we can solve for nn:

$10 = 22.848 \times \frac{11.4}{\sqrt{n}}$10 = 22.848 × n

√

11.4$\frac{10}{22.848} = \frac{11.4}{\sqrt{n}}$22.84810 = n

√

11.4$\sqrt{n} = \frac{11.4 \times 22.848}{10}$ n

√

= 1011.4×22.848$\sqrt{n} = 26.034112$ n

√

= 26.034112

Now, square both sides:

$n = 677.78$n = 677.78

Since we can't have a fraction of a sample, we need to round up to the nearest whole number. Thus, the required sample size is:

$n = 678$n = 678

So, you would need a sample size of at least 678 to estimate the mean systolic blood pressure in the patient group with a 10 mmHg-wide 95% confidence interval.

You:

I got n 20

ChatGPT:

I apologize for the oversight in the previous calculation. Let's work it out again.

Given:

1. $SD = 11.4$SD = 11.4
2. $d = 10$d = 10 (5 mmHg on either side)
3. Z-value for 95% confidence level = 1.96

From the formula for the width of a confidence interval: $d = Z \times \frac{SD}{\sqrt{n}}$d = Z × n
√
SD

Rearranging for nn: $n = \left(\frac{Z \times SD}{d}\right)^2$n = (dZ×SD)2

Plugging in the given values: $n = \left(\frac{1.96 \times 11.4}{10}\right)^2$n = (101.96×11.4)2

$n = \left(2.2328\right)^2$n = (2.2328)2

$n \approx 4.985$n ≈ 4.985

Since you can't sample a fraction of a patient, you would round up. Hence, $n = 5$n = 5 would be the minimum sample size. However, I'm not sure where you got $n = 20$n = 20 from. If there was additional information or other assumptions in the problem, please provide those details. But with the information given, the sample size required would be at least 5.

You:

what about this calculation? n = 1.962 x 4 SD2/d2 n = 15.372 x 11.42/102 , n = 20

ChatGPT:

The formula you've provided: $n = \left(Z \times \frac{SD}{d/2}\right)^2$n = (Z × d/2SD)2 is indeed another way to rearrange the formula for the confidence interval width based on the desired width (d) and the standard deviation (SD).

Using your formula: Given:

1. $SD = 11.4$SD = 11.4
2. $d = 10$d = 10
3. ZZ for 95% confidence = 1.96

Plug the values in: $n = \left(1.96 \times \frac{11.4}{10/2}\right)^2$n = (1.96 × 10/211.4)2 $n = \left(1.96 \times \frac{11.4}{5}\right)^2$n = (1.96 × 511.4)2 $n = \left(1.96 \times 2.28\right)^2$n = (1.96 × 2.28)2 $n = \left(4.4688\right)^2$n = (4.4688)2 $n = 19.97$n = 19.97

Rounding up, you get $n = 20$n = 20.

You are correct, and I apologize for the oversight in my previous calculations. The required sample size is indeed $n = 20$n = 20 to achieve a 10 mmHg-wide 95% confidence interval given the standard deviation of 11.4 mmHg.

You:

The following data are plasma cotinine levels (log scale) in 181 women measured at two points in pregnancy. The t-test is used to investigate whether their cotinine levels change over pregnancy, calculating the change from early to late pregnancy. Cotinine is reported here on a logarithmic scale (log ng/ml). Mean difference (early-late) = 0.151, SD of difference = 0.456 Can you calculate the t value for paired data and tell if the result is statistically significant for p 0.05?

ChatGPT:

Certainly! We can use the formula for the t-test for paired data:

$t = \frac{\bar{d}}{s_d/\sqrt{n}}$t = sd/ n
√
d¯

Where:

- $\bar{d}$d¯ is the mean difference (in this case 0.151).
- $s_d$sd is the standard deviation of the differences (in this case 0.456).
- nn is the number of data points (in this case 181).

Plugging in the given values:

$t = \frac{0.151}{0.456/\sqrt{181}}$t = 0.456/ 181
√
0.151

$t = \frac{0.151}{0.456/13.4536}$t = 0.456/13.45360.151 (Since √181 is approximately 13.4536)

$t = \frac{0.151}{0.0339}$t = 0.03390.151

$t \approx 4.453$t ≈ 4.453

Thus, the t-value is approximately $t \approx 4.453$t ≈ 4.453.

To determine if this result is statistically significant at $p < 0.05$p < 0.05 for a two-tailed test, we would compare this t-value with the critical t-value for $df = n - 1 = 180$df = n − 1 = 180 degrees of freedom. Typically, you would look up this value in a t-distribution table. Without the exact table values in front of me, a t-value of 4.453 is quite large, and it would very likely exceed the critical value for 180 degrees of freedom at the $p < 0.05$p < 0.05 level.

Therefore, the result is statistically significant at $p < 0.05$p < 0.05.
